# Draft Whole-Genome Sequence of the Purple Nonsulfur Photosynthetic Bacterium *Rhodopseudomonas rutila* R1

**DOI:** 10.1128/MRA.01267-18

**Published:** 2018-10-18

**Authors:** Sydney Robertson, Amiera Rayyan, Terry Meyer, John Kyndt

**Affiliations:** a College of Science and Technology, Bellevue University, Bellevue, Nebraska, USA; b Department of Chemistry and Biochemistry, University of Arizona, Tucson, Arizona, USA; Georgia Institute of Technology

## Abstract

Rhodopseudomonas species are purple nonsulfur bacteria found in many environments and known for their diverse metabolic capabilities. Here, we report the genome sequence of Rhodopseudomonas rutila type strain R1 and a whole-genome nucleotide comparison of related Rhodopseudomonas palustris species, suggesting the necessity for future reevaluation of the Rhodopseudomonas species differentiation.

## ANNOUNCEMENT

Rhodopseudomonas rutila is a Gram-negative purple nonsulfur α-photosynthetic bacterium that was originally described by Akiba et al. in 1983 (1). A later analysis by Hiraishi et al. (2) using phenotypic and chemotaxonomic comparisons indicated a close relationship to the Rhodopseudomonas palustris species, with DNA-DNA hybridization homology of 78% to the R. palustris type strain 2.1.6. As a consequence, R. rutila is currently considered a synonym for R. palustris. Although R. rutila and R. palustris are very similar in their microbiological properties, a study of the soluble electron transfer proteins of R. rutila described a cytochrome pattern that is significantly different from that of R. palustris 2.1.6 (3). To further clarify the taxonomic position of R. rutila, we sequenced the whole genome of the type strain R1.

The original R. rutila R1, isolated by Akiba et al., was obtained directly from the author shortly after its publication (1). We isolated DNA from frozen cells using the GeneJET DNA purification kit (Thermo Scientific). The DNA quantity and quality were determined using Qubit and NanoDrop and showed a ratio of absorbance at 260 nm to that at 280 nm of 1.74. The DNA library was prepared following the Nextera DNA Flex library prep kit instructions (Illumina). The genome was sequenced using 500 µl of a 1.8-pM library in an Illumina MiniSeq system with a paired-end library (2 × 150 bp), which generated 2,232,773 reads, yielding a total of 674.3 Mbp. Coverage exceeded 100×, which complicated assembly using Velvet version 1.2.10 (4). We therefore performed a random subsampling using the FastQ toolkit version 2.2.0 with a 75% sample read cutoff. The subsampled data set (1,674,579 reads) was assembled successfully *de novo* with Velvet. Velvet assembly used a minimum k-mer size of 21 and a maximum k-mer size of 121, and reverse complement reads were included. The assembled genome consisted of 149 contigs, with the largest contig being 354,499 bp with an *N*_50_ value of 90,178 bp. The GC content was 64.9%. The genome sequence was annotated using RAST version 2.0 (5), which indicated that R1 was 5,313,123 bp in length and that 5,090 coding sequences (CDs) and 49 RNAs were present. R. rutila has a complete set of Nap, Nir, Nor, and Nos genes for denitrification, as well as genes for nitrogen fixation.

Several nominal Rhodopseudomonas strains have been sequenced since 2004 (6–9). When we performed a JSpecies comparison (10) of the average percentage nucleotide identity between R. rutila R1 and published Rhodopseudomonas genome sequences, the following species/strains showed the highest identities: CG009, 97.4%; TIE, 97.4%; ELI1980, 97.2%; YSC3, 92.5%; PS3, 92.4%; 420L, 88.5%; DX1, 88.3%; pentothenatexigens, 88.3%; thermotolerans, 88.2%; XCP, 88.1%; AAP120, 82.1%; HA2, 81.8%; B29, 81.2%; and B5, 81.2%. The R. rutila R1 genome is related most closely to Rhodopseudomonas strains CG009, TIE, and ELI1980, which indicates that they are members of the same species. However, all the other Rhodopseudomonas genomes are below the 95% species cutoff, including the Rhodopseudomonas palustris type strain 2.1.6. As we recently noted with the Rhodopseudomonas sp. XCP genome (9), the low average nucleotide identity (ANI) between the various Rhodopseudomonas strains suggests that there is a need to revise and restructure the taxonomy. Based on our current analysis, strains R1, CG009, TIE, and ELI1980 all belong to R. rutila, while only strains 2.1.6 and B5 remain within the confines of the R. palustris species.

### Data availability.

This whole-genome shotgun project has been deposited at DDBJ/ENA/GenBank under the accession number QWVU00000000. The version described in this paper is version number QWVU01000000. The raw sequencing reads have been submitted to SRA under the accession number SRR7819324.
